# ICU nurses´ lived experience of caring for adult patients with a tracheostomy in ICU: a phenomenological-hermeneutic study

**DOI:** 10.1186/s12912-022-01005-x

**Published:** 2022-08-04

**Authors:** Abder Rahim Akroute, Berit Støre Brinchmann, Anders Hovland, Sven-Tore Dreyer Fredriksen

**Affiliations:** 1 Department of Anesthesia and Intensive Care Medicine, Nordland Hospital, N-8049 Bodø, Norway; 2 Faculty of Nursing and Health Sciences, Nord University, 8026 Bodø, Norway; 3 Nordland Hospital, 8076 Bodø, Norway; 4 Department of Cardiology, Nordland Hospital, Bodø, Norway; 5 Department of Clinical Medicine, UiT, The Arctic University of Norway, Tromsø, Norway; 6 Department of Health and Care Sciences, Faculty of Health Sciences, UiT, The Arctic University of Norway, Tromsø, Norway; 7 Campus Harstad, Havnegata 5, 9480 Harstad, Norway; 8 Huntington network, Knorrebakken 2, 9411 Harstad, Norway

**Keywords:** Caring, Intensive care unit nurses, Lived experience, Intensive care unit, Phenomenological-hermeneutic, Tracheostomy

## Abstract

**Background:**

The care of adult patients with a tracheostomy in intensive care unit is complex, challenging and requires skilled intensive care unit nurses. ICU nurses’ live experience is scarcely known. This study aimed to describe the lived experience of intensive care unit nurses of caring for adult patients with a tracheostomy in intensive care unit.

**Methods:**

This study employs a qualitative design. In-depth interviews were conducted with a purposive sampling of 6 intensive care unit nurses from a medical-surgical ICU of a university hospital in Norway who were interviewed. Data was analyzed and interpreted using a phenomenological-hermeneutic approach. This study was reported according to the Consolidated Criteria for Reporting Qualitative Research (COREQ).

**Results:**

The interpretation yielded the following themes and subthemes: 1) theme: ‘challenges of caring for patients with a tracheostomy’ consisted of the sub-themes: ‘difficult to communicate/interpret and understand the patient’s different forms of expression’, ‘complicated professional assessments’, ‘caring with patience’, and ‘collaborating with patient regarding challenges. 2) theme: ‘the satisfaction from providing care to patients with a tracheostomy’ consisted of the sub-themes: ‘working with intensive care patients is instructive’ and ‘importance to motivate’.

**Conclusions:**

ICU nurses experienced ambivalent feelings while caring for adult patients with a tracheostomy in ICU. They perceived caring as demanding owing to communication and collaboration at the same time, they experienced satisfaction while they strived to provide proper care and motivation. The identified challenges would lead to further improvement in nurses’ experiences and, in turn, the quality-of-care for patients with a tracheostomy. Awareness of these challenges is crucial to understand the need for an effective communication strategy to improve the quality and safety of adult patients with tracheostomy in ICU.

## Background

Tracheostomy in the intensive care unit (ICU) is one of the oldest known surgical procedures performed by creating an artificial opening in the anterior aspect of the neck, into the trachea [1]. A growing trend internationally has been increased use of tracheostomy for prolonged mechanical ventilation of patients in intensive care units [2]. According to Toker [3], tracheostomies may be temporary or permanent; short-or long-term, depending on the reason for its use. A short Survey reported that a substantial proportion of patients might have the tracheostomy, for several weeks or months, despite not requiring mechanical ventilation [4].

Caring for patients who require long-term admission to the ICU is both demanding, challenging, and complex [5]. It requires responsible, competent, and confident nurses to help save and secure patients from tracheostomy’s complications such as accidental decannulation, infection or a blocked tracheostomy tube. It has been widely recognized that nurses who work in general units, and even within ICU, can lack the necessary experience, knowledge, or confidence to provide safe and effective care for mechanically ventilated patients with a tracheostomy [6].

Despite the large number of international publications over the last decade [7–9] relating to tracheostomy care, there is still a paucity of research data from studies which explicitly investigated or described ICU nurses’ lived experiences of caring for adult patients with a tracheostomy in ICU. A qualitative perspective using the French Philosopher Paul Ricoeur’s [10] phenomenological- hermeneutic approach was chosen for this study because it is concerned with reaching a new understanding of the meaning of the phenomenon (caring for adult patients with a tracheostomy) as experienced by ICU nurses in the ICU. The implementation of a phenomenological-hermeneutical perspective allows the ICU nurses to describe their experience of caring for adult patients with a tracheostomy in ICU.

Furthermore, the aim of this study is to address this gap by describing ICU nurses’ lived experiences of caring for adult patients with a tracheostomy in ICU. The results can contribute to a deeper understanding of ICU nurses’ responsibility in caring for patients with a tracheostomy and to better caring practice for patients in ICU. Therefore, it seems important to take a close look at the phenomenon of caring for adult patients with a tracheostomy in ICU from the perspective of ICU nurses. The aim of this study is to highlight and interpret the meaning of ICU nurses’ lived experience with caring for adult patients with a tracheostomy in the ICU.

## Methods

### Study design

This is a phenomenological-hermeneutic approach inspired by Ricoeur [11] and developed by Lindseth & Norberg [12]. A lifeworld approach is advisable to gain a deeper understanding of care through investigating ICU nurses’ perspectives on the phenomenon of caring for adult patients with a tracheostomy in the ICU [13]. Ricoeur [14] stated that a phenomenological-hermeneutic understanding of self emerges through narratives and offer new insight into the self. Ricoeur [14] maintains that there exists a reciprocal relationship between phenomenology and hermeneutics. Phenomenology focuses on the content of a person’s perceived experience and hermeneutics entails interpretation of the text of an interview, to reveal the actual meaning of a person’s experience [12–14]. These philosophies are equally important in order to reach a comprehensive understanding of text [14]. The interpretation process is not linear in a phenomenological-hermeneutic interpretation method. The researchers followed the text’ movement, between the whole text and its parts in a hermeneutic circle to strengthen the argument for a trustworthy interpretation of the narratives [15].

### Setting and participants

A total of 6 rotation-shift ICU nurses working permanently in a medical-surgical ICU from a university hospital in Norway took part in the study. The inclusion criteria for participants in this study included experienced ICU nurses providing care to adult patients with a tracheostomy in ICUs with no less than 5 years of nursing experience. The first author, who works as an ICU nurse from a different hospital with no relationship to the participants in this study, sent e-mails to the head nurse, including information about the study and a request for participants who had experience with caring for adult patients with a tracheostomy in ICU. 8 ICU nurses responded to the researcher directly with confirmation of participation in the study. The ICU nurses were informed that participation was voluntary and were provided written and oral information about the research project and asked to sign a consent form prior to participation. 6 ICU nurses accepted to participate (Table 1), and 2 additional ICU nurses expressed interest but did not respond to a follow-up inquiry to schedule an interview. The informants were interviewed in a quiet space in the hospital.

**Table 1:** Demographic characteristics of the participants

Participant’s code	Gender	Age (years)	Work experience (years)	Level of education	Position
**1**	**female**	**45**	**20**	**. Bachelor’s degree. ICU nurse**	**ICU nurse**
**2**	**female**	**38**	**12**	**.Bachelor’s. Degree. ICU nurses.**. M**Sc**	**ICU nurse**
**3**	**female**	**57**	**32**	**.Bachelor’s degree.**. I**CU nurses.. MSc**	**ICU nurse**
**4**	**female**	**31**	**12**	**. Bachelor’s degree.. ICU nurses**	**ICU nurse**
**5**	**female**	**58**	**35**	**.Bachelor’s degree.. ICU nurse**	**ICU nurse**
**6**	**female**	**33**	**11**	**. Bachelor’s degree.. ICU nurse**	**ICU nurse**

### Data collection

Data were collected from the beginning of October to the end of November 2019. The main researcher (AA) collected data by using in-depth, open-ended interviews that encouraged the informants to express their narratives, thereby providing a deeper understanding of their lived experiences [16]. They were interviewed individually and asked to describe their experiences with the key question in the study: ‘can you talk about your experiences with caring for adult patients with a tracheostomy?’ Subsequently, the interviewer expanded the interview to investigate their experiences by adding questions or statements such as: ‘What do you mean’, ‘Please explain’, could you be more specific?’ Each interview lasted approximately 60 minutes and was audiotaped with the ICU nurses’ permission. The tapes were transcribed verbatim (word by word), and translated to English, including all emotional expressions, for a more accurate reflection of the informants’ experience [12]. Data collection and analysis continued until theoretical saturation was reached, so that no new substantive information was collected.

### Ethical considerations

Consistent with The Norwegian legislation, collecting data about professional healthcare workers job experiences has to be assessed ethically by Data Protection Official for research at the Norwegian Social Science Data Services (NSD). This was done in the current study (reference number 963088). NSD confirmed that the study met the requirements for ethical soundness in relation to standards and codes of ethics. The study was performed according to the Declaration of Helsinki. The ethical principles of autonomy, beneficence, non-maleficence, and justice were assured following the Helsinki declaration. The ICU nurses were given written as well as oral information and written informed consent was obtained from all ICU nurses. ICU nurses were informed that they could withdraw from the study at any time.

### Data analysis

Since this is a phenomenological-hermeneutic study of lived experiences of ICU nurses caring for adult patients with a tracheostomy in ICU, an interpretive process was considered appropriate. The researchers (AA and STDF) applied Lindseth and Norberg’s [12] phenomenological-hermeneutic analysis. This approach is useful to illuminate and describe the meaning of lived experiences through interpretation of personal narratives. According to Ricoeur [10], a person’s lived experience remains private, but its meaning may be revealed through interpretation of a narrative. Interpretation of the texts constitutes a progression from understanding to explanation and from explanation to comprehension. In line with Lindseth & Norberg [12], the analytical process consists of a movement between three steps: naïve reading, structural analysis, and comprehensive understanding. The first step is *naïve reading* (initial reading). The transcribed interviews were read several times with an open mind and willingness to experience immediate impressions (naïve reading), disregarding the preconceived understanding of the interviewer. The analysis moves towards the phenomenological world, allowing researchers to be touched by the narratives. According to Ricoeur [10], the researchers move from the natural approach into a phenomenological approach with the naïve reading, which reflections on actual on meaning of the text. The naive understanding of the text suggests the direction for the structural analysis [14]. The second step is *structural analysis* (i.e., decontextualization of the text) and searches for sub-themes or themes. The text was divided into meaningful units, comprising “what was said” in the text and reformulated to “what was meant” in the text, then condensed, reflected on, sorted according to similarities and differences, and finally separated into sub-themes and themes. The objective of the structural analysis was to explain what the text was saying. The sub-themes and themes are presented in the results section. The last step *comprehensive understanding* (i.e., re-contextualizing of text) is a critical reading leading to comprehensive understanding which was devised as a critical, in-depth interpretation, following the text from what it said to what it talked about. The naïve understanding, the findings of the structural analysis, the authors’ pre-understanding, previous research results and relevant theory were all brought together in the comprehensive understanding, that aimed at a deeper understanding of the studied phenomenon [12]. The narratives were analyzed by the first and second author independently and findings jointly discussed, from the naïve reading to the structural analysis and finally comprehensive interpretation. The researcher’s background as a practicing ICU-nurse may also influence the data/results in this study. However, the researcher has kept in mind bracketing, reflected on, and tried to avoid personal assumptions in all the writings. The second author was involved in the analytical process, thereby increasing its objectivity. The naïve reading, structural analysis and comprehensive understanding are presented under results.

### Trustworthiness

To ensure trustworthiness, the concepts credibility, confirmability, dependability, and transferability [17] were discussed throughout the study process. 1) Credibility was obtained through the researcher’s long-term involvement in the study, and by employing a qualitative method which is well-established, and by using ICU nurses who freely volunteered their time, thus ensuring that they were genuinely interested in providing data freely. The study and data analysis process were diligently executed by the research team. For this purpose, the two authors (AA and STDF) performed coding of the texts independently and met to discuss any discrepancies establishing and achieve consensus about the initial coding and emerging themes and subthemes. 2) In order to ensure confirmability, critical discussions were conducted between the authors. Two faculty members specializing in qualitative research methods confirmed the data analysis process. Rich description of findings, and conformation of findings by three non-participating ICU nurses helped to ensure data transferability. 3) In order to secure the dependability of the data, the same researcher who conducts the interviews transcribes the data verbatim. 4) In order to ensure transferability, the researchers tried to provide an accurate report of ICU nurses statements that can be used in other contexts.

## Results

This qualitative study was based on in-depth and open-ended interviews with 6 female ICU nurses who were permanently employed at a medical-surgical ICU. Their ages ranged between 31 and 58 years, and their years of experience from the ICU ranged between 11 and 35 years. All participants had bachelor’s degree in nursing and were ICU- nursing’s specialists. Two participants had Master of science in Nursing.

### Naïve understanding

Each interview was listened to and read several times to gain an impression of the whole. The re-reading and the understanding of the whole text means to be moved by the text and to gain an idea and guess what the text is about. The reading showed that ICU nurses experienced different challenges while caring for adult patients with a tracheostomy in ICU. They encountered difficulties in communication, and collaboration with adult patients with a tracheostomy. ICU nurses reported that they experienced frustration and those patients were treated aggressively and sometimes it was hard to maintain motivation. At the same time, ICU nurses reported that caring for adult patients with a tracheostomy was satisfying.

### Structural analysis

The next step when entering into the structure analysis was for the authors to validate our guesses and further explain the meanings within such phenomena as caring for adult patients with a tracheostomy in ICU. The results of the structure analysis were thus presented in 2 themes and 6 sub-themes highlighting the significance of ICU nurses’ lived experiences from caring for patients with a tracheostomy in an ICU. The findings will be presented in the order set out in Table 2.

**Table 2:** Sub-themes and themes constructed from the analysis of the interviews

Sub-themes	Themes
Difficult to communicate/interpret and understand the patient’s different forms of expression	Caring for adult patients with a tracheostomy is challenging
Complicated professional assessments	
Caring with patience	
Collaborating with patient regarding challenges	
Working with intensive care patients is instructive	Satisfaction from providing care to adult patients with a tracheostomy
Importance of motivation	

### Caring for adult patients with a tracheostomy is challenging

In this qualitative study, ICU nurses identified diverse challenges while providing care for adult patients with a tracheostomy in the ICU.

#### Difficult to communicate/interpret and understand the patient’s different forms of expression

The narratives revealed that caring for adult patients with a tracheostomy was demanding and challenging. ICU nurses reported that communication represented a major challenge. It is well-documented that tracheostomy affects voice production, leaving patients unable to communicate their needs to the nurses. The communication difficulty causes distress and frustration, especially, when patients were unable to speak. Two ICU nurses pointed out the following,

*“The biggest challenge is communication. It is positive and negative; the positive is that you find creative ways to communicate, and the negative is that it is exhausting...yes … for nurses … I don't understand what they want”.* (ICU nurse 3)

Another ICU nurse stated the following.

*“I mean, the hardest thing is that they don’t express themselves enough, and the patient gets frustrated because we don't understand what they're trying to say, I feel … you can see their frustration*”. (ICU nurse 5)

This can be read as that the ICU nurse’s biggest challenge is to understand the patient. Patients may be unable to vocalize their needs due to the tracheostomy, may be sedated or have fluctuating consciousness. Some patients were not able to write due to swollen hands/fingers, loss of physical strength or lack of coordination. The ICU nurses saw both challenges and opportunities in the communication situation. This can be interpreted as the ICU nurses being creative in finding better ways to communicate with patients and trying different methods to be able to understand, such as interpreting body language, using picture word-phrase boards or tablet applications designed for patient communication.

#### Complicated professional assessments

ICU nurses expressed frustration, dissatisfaction, and concern about overtreatment of patients with a tracheostomy in ICU. One ICU nurse said:

*“Sometimes, you find yourself thinking that it’s not just people with a tracheotomy, but … we are treating people who are dying, that the natural …. I mean, sometimes we overtreat people”.* (ICU nurse 1)

It seems that the ICU nurses experienced that adult patient with a tracheostomy were being treated intensively and reported that it was difficult to know where the boundaries for over- or under-treatment lie. They reflected that it was difficult to clearly define the usefulness of the treatment. Providing care was difficult when it had no apparent benefit to the patients. ICU nurses claimed that witnessing patients’ suffering was painful. They reported that the combination of patient’ comorbidity, age, cognitive impairment, and the continuation of futile treatment of long-term critically ill adult patients with a tracheostomy in ICU impacts not only on that patient, potentially prolonging suffering, and the natural dying, but also on family and ICU nurses. ICU nurses experienced the futile care as frustrated. One ICU nurse said:

*“Yes, but … can’t they just … let life, in a way, take its own course...Instead of being in intensive care for a month, so...you know almost certainly that if you are that critically ill, and you are 90 years old … then...yes, well … .fortunately, it’s not us who decide* ”. (ICU nurse 4)

When ICU nurses claim that they don’t see the positive side of certain treatments in the ICU, it can be understood that, in their opinion, some patients are over-treated. They wonder when the treatment will end. It seems difficult to maintain motivation when they have full focus on nursing and the situations remains constant over time. ICU nurses reported experiencing burnout and sadness. They placed great emphasis on the challenges of witnessing the decline in patient’s needing long-term intensive care or seeing lack of progress. ICU nurses saw their task as helping patients and pointed at feeling frustrated and sad when patients did not progress or get better under their care.

#### Caring with patience

ICU nurses experienced that some of the adult patients with tracheostomy were demanding, but at the same time they said that one should remember to be professional and satisfy the patient’s needs in a proper way. One ICU nurses said the following:

*“It’s certainly a challenge. Because of … the patients’ insecurity, especially those who are mechanical ventilated … and still not being fully conscious. And then giving them a sense of security and helping them to understand that they are in safe”*. (ICU nurse 3)

The narrative from the ICU nurse can be understood as focusing on the patients’ safety and well-being. It seems as if there are special situations the nurse refers to that are particularly challenging, for example when patients are connected to artificial ventilation and comatose, yet still seeking safety because patients were stressed and scared. Another ICU nurse said:

*“It’s a balancing act...in some way, the patient is demanding, always needing their pillow adjusting an inch, and yes … That’s how it is, in most cases”****.*** (ICU nurse 5)

It seems that ICU nurses experience a balancing act in demanding situations with severely ill patients. The ICU nurses’ underlying attitude is to care for the patients in the best possible way, and with dignity, which requires patience and balance.

#### Collaborating with the patient regarding challenges

ICU nurses reported that adult patients with tracheostomy were in a challenging situation and suffered from chronic obstructive pulmonary disease (COPD). This made it more difficult for patients and ICU nurses to work together. One ICU nurse told us that:

*“There are often such demanding patients since they are conscious and many of them have COPD … many of them are anxious and stressed. Weaning from mechanical ventilation is often challenging, and it can be difficult to get the patient to be cooperative”*. (ICU nurse 4)

The ICU nurses experience can be understood as being difficult to relating to patients have who suffer from COPD. This is often accompanied by anxiety and stress reactions related to weaning from artificial ventilation. It seems as if these reactions also affect the patients, which again influences collaboration with the staff.

They also pointed out that some patients become delirious, which makes communication more difficult. An example from one of the ICU nurses who said:

*“If they are delirious, it’s even harder for them to talk. You don’t know if they’re trying to tell you something, or if they are seeing things or believing themselves to be somewhere else. Communication is certainly a challenge”.* (ICU nurse 6)

It may seem that when patients are also affected by ambiguity in relation to time and place, their confusion increases. This complicates the situation for the ICU nurses in terms of understanding and perception of the patient’s concern.

### Satisfaction from providing care to adult patients with a tracheostomy

The narratives demonstrated that ICU nurses expressed satisfaction while caring for patients with a tracheostomy in ICU, and that their encounter with patients was exciting and instructive.

#### Working with intensive care patients is instructive

The narratives about caring for adult patients with a tracheostomy at the ICU and that their meetings with patients’ relatives were important and informative. One ICU nurse reflected about this and said:

*“You get to know the patient and their relatives. It’s very rewarding to work with critically ill patients. You meet them when they are vulnerable, I’d say. It’s good to help them to get better, to see that they can leave from ICU”.* ( ICU nurse 2)

It may seem to ICU nurses that working with patients with tracheostomy and their relatives takes place during a critical and vulnerable phase. ICU nurses also seem to express a general satisfaction with the very challenge associated with helping, but also with the fact that patients recover and can leave the ICU. The story of one of ICU nurse shows how this satisfaction is expressed among the nurses:

*“The best is when they come back to visit us … a patient you have had over a long period, you become engaged with. And you always want him to go well, and when it does … after a month or two, maybe three or four, they come back to visit us. I think that’s the loveliest thing that can happen. And then you are rewarded, in a way, for all you have done*”. ( ICU nurse 6)

The example described can be suggest that when patients have recovered, they come back to visit the ICU. It seems that ICU nurses greatly appreciate such visits. It is a form of ‘moral reward’ for their efforts and the care they have provided.

#### Importance of motivation

ICU nurses told us how they provided motivation to patients and their relatives for different activities they knew would work, but which also required some prerequisites. An ICU nurse said:

*“You can get good contact with the relatives, you get to know them well when the patient is conscious, and you care for them for a long time. You know what works … and so there is mobilization of the patient every day and you can see progress being made”.* (ICU nurse 3)

ICU nurses seem to understand what works for patients with a tracheostomy, and what motivates them. With this knowledge, they motivate the patients in different ways every day, and they also observe progress in the patients every day. ICU nurses viewed the patient and relatives as a resource in care. They also tried to involve and engage relatives in the care for their loved ones and actively used them in the treatment. An ICU nurses said:

*“One sees them as a resource....one use them in the treatment … work together. When they are visiting ICU. I try to use them as much as possible, because I believe that they also think that it’s good for them … better … instead of just sitting there and feeling completely helpless in the situation”.* (ICU nurse 1)

Using relatives in the treatment process can be read to imply that in ICU nurses experiencing, relatives felt good with their loved ones when they were encouraged to participate in the care rather than sitting still and feeling helpless. ICU nurses commented on the positive feeling they experienced when patients and relatives appreciated the effective care they provided.

## Comprehensive understanding and reflections

The entire text was read as a whole in the last phase of interpretation. The naïve understanding, the results from the structural analysis, the authors’ pre-understanding were brought together and reflected in light of the literature in order to reach a new comprehensive understanding [12]. The aim of the study was to describe ICU nurses’ lived experience of caring for adult patients with tracheostomy in ICU. The ICU nurses experienced both challenges and feeling of satisfaction. The comprehensive understanding and reflections were based on the main findings: caring for adult patients with a tracheostomy is challenging and satisfaction from providing care to adult patients with a tracheostomy.

The analysis showed that ICU nurses experienced difficulties in interpreting the patients’ message. They had to be creative and sometimes act spontaneously to meet the needs of voiceless patients, which leaves ICU nurses to learn communication strategies through trial and error [18]. However, ICU nurses emphasized that misinterpretation often prevented patients with a tracheostomy from expressing their opinions and needs, causing distress and frustration both to patients and ICU nurses. ‘Frustration’ has previously been used as a term to characterize difficulties with communication in the ICU, shared reaction among patients and nurses [19, 20]. Consistent with recent research [21], this study reports that ICU nurses feel stressed when trying to understand voiceless patients with tracheostomy who are unable to verbally express their symptoms, pain levels or needs.

The findings in this study further suggests that ICU nurses found it difficult to understand or read the lips of patients with inflated trachea cuff because they were unable to use their voice, and many were unable to speak for an extended period of time. These patients found it difficult to express basic needs and desires and difficulty to participate in life and death decisions, despite effective communication techniques and tools for patients with tracheostomy; such as communication kits, pictorial symbols, lips reading, eye contact, note-pads, or writing [18], that are considered essential for accurate and effective assistance to patients. Previous research demonstrates that nurses currently make little or no use of these devices for patients in ICU [22, 23]. Furthermore, ICU nurses emphasized that communication with patients with a tracheostomy was both challenging and exciting. Consistent with recent studies [24, 25] that showed health care personnel describing the experience of caring for a patient who is conscious on artificial ventilation as ‘demanding yet rewarding’. The inability to effectively communicate makes it difficult for patients to understand and be active participants in their treatment, and miscommunication between ICU nurses and patients may lead to poorer patient care, unnecessary suffering and dissatisfaction, and longer hospitalization [26].

The findings in this study also highlight another challenge that ICU nurses experienced, namely disagreement about the unreasonably intense medical treatment of critically ill adult patients with tracheostomy in ICU. ICU nurses experienced intense moral distress and were obviously concerned about the perceived inappropriate and unnecessary suffering of patients due to excessive treatment. Our findings are in accordance with previous research [27, 28]. Unfortunately, this study showed that patients with tracheostomy received excessive care, which presented a substantial ethical problem for the ICU nurses. A recent study also demonstrates that unnecessary life-sustaining treatment has important ethical implications and violates the four fundamental principles of ethics. It disregards the autonomy, dignity, and integrity of the patient and excessive life-sustaining treatment increases pain and suffering, prolongs the dying process, increases the relatives’ suffering, and causes frustration among caregivers [29]. Although it frustrated ICU nurses to witness patients ‘suffering due to treatment they disagreed with, they still provided treatment according to instructions. Other studies have highlighted disagreements and communication problems between health care personnel in the ICU; regarding the decision-making process, failure to address the futility of a treatment, initiation of end-of-life discussions. Also, that collaboration between doctors and nurses was one of the major challenges in ICU care [30, 31]. The most important negative consequences of initiating futile treatments are increased suffering for patients, excessive costs, moral distress, burnout, job dissatisfaction, and increased turnover among nurses and physicians [32].

As our findings demonstrates, caring for adult patients with a tracheostomy necessitates a certain level of patience. Literature describes that the ICU environment is stressful and ICU patients often experience anxiety, fear, dyspnea, powerlessness, and lack of control. These patients also suffer from an inability to communicate [33]. Consequently, bedside ICU nurses play a fundamental role to patient safety and possible deterioration with continuous clinical examinations [34]. ICU nurses found patients with tracheostomy demanding. However, ICU nurses live up to their role, providing help to overcome anxiety and fear, and creating a positive environment with a sense of value and motivation for the patient. ICU nurses reported that they tried to provide comfort and to enable patient involvement and support. The need for ICU nurses to be patient’s advocate cannot be overemphasized. Like in another recent study [35], nurses can empower patients, deliver them from discomfort and unnecessary treatment, protect them from malpractice through advocacy. Moreover, the present study reports that ICU nurses provide compassionate care. Previous research confirms that nurses act with patience, provide compassionate care, are dedicated and empathic towards their patients [36, 37].

Working with adult patients with tracheostomy and chronic obstructive pulmonary disease (COPD) was stressful, due to the complexity of the disease. ICU nurses found it challenging to comfort, strengthen, support, and encourage patients with COPD while also handling equipment. For patients with a tracheostomy and severe COPD, breathlessness is a major problem and represents a challenge to both ICU nurses, patients, and their relatives. ICU nurses reported that it was difficult to wean patients with COPD off from artificial ventilation. Failed weaning is a physical and psychological strain on patients as well as ICU nurses, which complicates the collaboration between nurses and patient. Previous research has demonstrated that psychological distress is significantly higher and common among patients with COPD [38]. Therefore, ICU nurses need to create encounters which promote positive experiences of care for patients with COPD. This group of patients depend to nurses’ openness, patience, empathy, communication, and sensitivity [39].

ICU nurses report that it is very difficult to collaborate with delirious patients with tracheostomy. Certain patients were at high risk for developing delirium because they were older and had pre-existing cognitive impairments, electrolyte disturbance, frequently by ICU medications such as benzodiazepines. Benzodiazepines are among the key factors associated with delirium and common in ICU [40]. ICU nurses experienced difficulties with understanding their patients and in satisfying their needs. Consistent with the findings in a recent study [41], which reported that ICU nurses emphasize the impact of heavy workloads with caring for confused long-term intensive care patients with tracheostomy, and a sense of insufficient capacity to fulfil their role satisfactorily. They pointed out that patients with delirium were time consuming, in need of significant direct medical costs, resources and nursing. Moreover, caring for patients with delirium generates stress, anxiety, and emotional conflicts for ICU nurses.

ICU nurses’ interactions with adult patients with tracheostomy and their relatives were positive and instructive. This study demonstrates how ICU nurses found satisfaction from caring for them, especially the most vulnerable and most in need. This is consistent with previous research showing that nurses gain a sense of compassionate satisfaction, which is defined as ‘the positive feelings derived from helping others through traumatic situations patients and the satisfaction that derives from ‘making a difference’ in patient care, recognition by management, and appreciation from patients and relatives [42]. They experienced happiness when patients were discharged from the ICU to wards, which usually meant that the patient was well on the way to recovery from serious illness. Moreover, ICU nurses emphasized the importance of family meetings as a significant forum to get to know the patient better, to discuss the patient’s condition, prognosis, and care preferences. They appreciated relatives’ visiting their loved ones and involvement in treatment. Previous studies [43] point to the relatives’ desire to be there for the patient, but generally relatives have been considered visitors more than as a resource for patients [44]. Knowing that a family member is at the patient’s bedside relieves some of the ICU nurse’s burden.

This study confirms the importance of motivating patients with tracheostomy and their relatives. ICU nurses spoke of the responsibility to motivate and to restore the patients’ self-confidence by helping them to accept the situation, and gather the necessary strength required to improve and find energy, enthusiasm, and determination. This comparable to the findings in a recent study [45] which points to patient empowerment in the ICU consisting of strengthening and stimulating the patients’ own inherent joy of life, will-to fight, and a positive environment of reassurance, strengthening feelings of value and motivation, in which the patient felt safe, was physically well cared for, was encouraged to participate, and received all necessary information. This study confirms that relatives were a resource to both patients and ICU nurses. The presence of relatives constituted an important source of psychological stability for the patient, as well as a source of support for better recovery. This is in line with a study [46] that reports that relatives are a resource to ICU nurses as well as patients. Therefore, enhancing relatives’ and patients’ motivation in the ICU is an important part of caring.

### Limitations and strengths

The study was carried out in only one university hospital with a small contingency of respondents, which potentially limits the transferability of the study. However, the ICU nurses’ experiences of caring and their willingness to share their experiences, provide sufficiently rich descriptions. It is not established that the richness of data correlates with the number of ICU nurses [47]. Data saturation was reached in the collected data. Consequently, the findings might also have some degree of relevance to other ICUs. The phenomenological-hermeneutic method [12] used in this study was a suitable method for discovering the meaning of lived experiences of ICU nurses caring for adult patients with tracheostomy in ICU. The participants in this study were chosen by purposive sampling, that is, the researchers selected the participants that fulfill the needs of the study; ICU nurses with 5 years of experiences. A major criticism of this type of sampling is that the sample is biased by the selection process. The participants in this study varied in age and experiences, and we believe that the sample was sufficient to gain richness in data. Moreover, we need more research about inexperienced nurses caring for adult patients with a tracheostomy. Finally, the organization, delivery and availability of intensive care services are not consistent across Norway and therefore, ICU nurses working in other urban or rural hospitals might have other experiences.

## Conclusion

This qualitative phenomenological-hermeneutic study provides a unique and detailed descriptions of ICU nurses’ ambivalent and lived experience of caring for adult patients with tracheostomy in ICU. Although ICU nurses experienced difficulties in communication and collaboration difficulties with these patients and were frustrated with the too excessive treatment these patients sometimes received, they recognized that they could address such difficulties by improving their communications skills. They realized that empathy and patience enabled meaningful communication and caring. The findings in this study strengthen the evidence that there is a need to have greater focus on improving the care of adult patients with tracheostomy in ICU, stronger focus on education, nurse-patient interaction, and nurse-physician communication. There is a need for stronger collaboration in the decision-making process as well as implementation of tracheostomy care guidelines in ICU. Furthermore, organizations should review staffing and education policies considering the factors which lead to suboptimal care for adults’ patients with a tracheostomy. Hence, it is of great importance for policy makers, managers to recruit nurses who have the characteristics, competencies and confidence required for providing proper care in ICU. Staff education of intensive care unit personnel is widely recommended, but further development is needed to determine the best methods of delivering education, especially for health care professionals who care for patients with a tracheostomy. The use of communication aids should be a prioritized field, as should the implementation of a variety of communication aids. More multidisciplinary approaches in future studies are required to enhance the best methods of promoting best practice to improve patients with a tracheostomy.

## Data Availability

The datasets analyzed during this current study is available from the corresponding author on reasonable request.

## Abbreviations

ICU: Intensive care unit
ICU nurses: Intensive care unit nurses
